# Methylenetetrahydrofolate reductase *C677T* polymorphism is associated with estimated glomerular filtration rate in hypertensive Chinese males

**DOI:** 10.1186/1471-2350-13-74

**Published:** 2012-08-16

**Authors:** Qing Dong, Genfu Tang, Mingli He, Yunqing Cai, Yefeng Cai, Houxun Xing, Liming Sun, Jianping Li, Yan Zhang, Fangfang Fan, Binyan Wang, Ningling Sun, Lisheng Liu, Xiping Xu, Fanfan Hou, Hongbing Shen, Xin Xu, Yong Huo

**Affiliations:** 1 Department of Epidemiology and Biostatistics, School of Public Health, Nanjing Medical University, Nanjing, China; 2 School of Health Administration, Anhui Medical University, Hefei, China; 3 Department of Neurology, First People’s Hospital, Lianyungang, China; 4 Department of Neurology, Guangdong Traditional Chinese Medicine Hospital, Guangzhou, China; 5 Lianyungang Center for Advanced Research in Cardiovascular Diseases, Lianyungang, China; 6 Department of Cardiology, Second People’s Hospital, Lianyungang, China; 7 Department of Cardiology, Peking University First Hospital, Beijing, China; 8 Institute of Biomedicine, Shenzhen University, Guangzhou, China; 9 Department of Cardiology, Peking University People’s Hospital, Beijing, China; 10 Division of Hypertension, Fu-wai Hospital, Beijing, China; 11 Division of Epidemiology and Biostatistics, University of Illinois at Chicago School of Public Health, Chicago, Illinois, 60642, USA; 12 Institute of Nephrology, Southern Medical University, Guangzhou, China

**Keywords:** *MTHFR C677T* polymorphism, eGFR, CKD

## Abstract

**Background:**

Plasma level of total homocysteine (tHcy) is negatively correlated with kidney function in general population. However, the causal mechanism of this correlation is poorly understood. The purpose of this study is to investigate the association of methylenetetrahydrofolate reductase (*MTHFR*) *C677T* gene polymorphism, which is a major genetic determinant of the plasma tHcy level, with estimated glomerular filtration rate (eGFR) in Chinese.

**Methods:**

A total of 18 814 hypertensive patients (6 914 males, 11 900 females) were included in the study.

**Results:**

Association between the eGFR and *MTHFR C677T* genotype was examined by sex-specific regression analyses. In males, TT genotype was associated with 1.37 ml/min/1.73 m^2^ decrease in eGFR (p = 0.004) and with an increased risk (OR = 1.32, p = 0.008) for the lowest quintile of eGFR after adjusting for age, BMI, and blood pressures. However, such association was not observed in females (p > 0.05). This association suggests *MTHFR C677T* polymorphism may play a role in the regulation of eGFR in males.

**Conclusions:**

*MTHFR 677 T* is a risk allele for decreased kidney function in Chinese males, implicating this gene in the pathogenesis of chronic kidney disease (CKD).

## Background

Glomerular filtration rate estimated from serum creatinine level (eGFR) is an important measurement of kidney function, and frequently used to define stages of chronic kidney disease (CKD). Plasma level of total homocysteine (tHcy) is negatively correlated with kidney function in general population [1,2]. However, the causal mechanism of this correlation is poorly understood. Significant loss of kidney function will inevitably lead to hyperhomocysteinemia, as frequently observed in patients with end-stage renal disease. On the other hand, hyperhomocysteinemia could induce glomerular injury in animal studies [3-7], though it’s not clear if hyperhomocysteinemia play a pathogenic role in decreased kidney function in humans. A recent prospective study suggested tHcy is an independent predictor for CKD [8].

Methylenetetrahydrofolate reductase (MTHFR) catalyzes the conversion of 5,10-methylenetetrahydrofolate to 5-methyltetrahydrofolate, a co-substrate for homocysteine remethylation to methionine. *C677T*, a single nucleotide polymorphism (*C– > T*) at nucleotide position *677*, leads to *Ala– > Val* codon substitution at amino acid position 222. *MTHFR C677T* is a major genetic determinant for hyperhomocysteinemia, and has been shown to be associated with risks for stroke, coronary heart disease, neural tube defect, depression, schizophrenia, cancer, and a number of other disease statuses [9-14]. However, few have investigated the association between *C677T* and kidney function. Given the fact that kidney function and tHcy is well correlated and that *C677T* is a major determinant of tHcy, it would be interesting to test if *C677T* is associated with kidney function in general populations.

In this report, we performed a cross-sectional analysis on association of *MTHFR C677T* polymorphism with eGFR in 18814 Chinese adults.

## Methods

### Study population

The study subjects were participants of an ongoing China Stroke Primary Prevention Trial (CSPPT, clinicaltrials.gov identifier: NCT00794885) in Lianyungang, Jiangsu province of China. CSPPT is a multi-center randomized controlled trial designed to confirm that enalapril maleate and folic acid tablets is more effective in preventing stroke among the patients with primary hypertension when compared with enalapril maleate. Details for inclusion/exclusion criteria, treatment assignment, and outcome measures of the trial were described elsewhere (http://clinicaltrials.gov/ct2/show/NCT00794885). In the current study, we included 18 814 subjects from Lianyungang who participated in the screening phase of the trial and had valid measurements of *MTHFR C677T* genotype and baseline serum creatinine. The Human Subject Committee at the Biomedical Institute of the Anhui Medical University approved the study.

### Data collection

Researchers went to the communities to screen local residents for hypertensive patients. Candidate hypertensive patients were then invited to study centers for a formal screening visit. Each participant was asked to fast after 10 PM the night before the visit. During the screening visit, every participant gave the informed consent and then the following baseline data were collected.

#### Questionnaires

Questionnaires were administered to collect information on sociodemographic status, occupation, diet, lifestyle, health behavior, medical history, and medication, as well as reproductive history for women.

#### Anthropometry

Height was measured without shoes to the nearest 0.1 cm on a portable stadiometer. Weight was measured in light indoor clothing without shoes to the nearest 0.1 kg. WC was measured as the minimum circumference between the inferior margin of the ribcage and the crest of the iliac. Hip circumference was measured at the level of maximum extension of the buttocks. BMI was calculated as weight/height squared (kg/m^2^).

#### Blood pressure

Resting blood pressures were measured three times in a sitting position after 10 minutes of rest using a mercury sphygmomanometer. Three blood pressure reads were then averaged.

#### Phlebotomy

Venous blood was drawn from the forearm of each participant in the fasting status. Serum and plasma were separated from blood cells in the field within 30 minutes and kept frozen at −70°C.

### Laboratory assays and GFR estimation

Serum creatinine was measured by a modified kinetic rate Jaffe reaction method using a Dade Dimension Chemistry Analyzer (Siemens, Germany). Serum glucose and total cholesterol were also measured on the same analyzer. Plasma homocysteine was measured by an enzyme-cycling method using a Hitachi 7020 Automatic Analyzer (Hitachi, Japan). DNA was extracted from leukocytes in peripheral blood using standard techniques. *MTHFR C677T* genotype was determined by Taqman assay designed and manufactured by Applied Biosystems (Foster City, CA).

EGFR was estimated using the CKD-EPI equation as following [15]: eGFR = 141 × min (Scr/κ,1)^α^ × max (Scr/κ,1)^-1.209^ × 0.993^Age^ × 1.018 [if female], where Scr is serum creatinine (mg/dL), κ is 0.7 for females and 0.9 for males, α is −0.329 for females and −0.411 for males.

### Statistical analyses

The empirical sex-specific distributions of eGFR were estimated using a kernel density estimating function. Genotype distribution was tested for Hardy-Weinberg equilibrium (HWE) using a χ^2^ test. Sex-specific regression analyses were performed to investigate the association between eGFR and *C677T* genotype and to estimate the genotype’s odds ratio for low eGFR and CKD, adjusting for age, BMI, and systolic and diastolic pressure. All the analyses were done using the statistical package R [16].

## Results

This study includes 18814 hypertensive subjects (6914 males, 11900 females) from Lianyungang, China who participated in the screening phase of CSPPT and had valid measurements of baseline serum creatinine level and *MTHFR* genotype. The phenotypic characteristics of subjects were summarized in Table 1. In this population females had considerably higher body mass index (BMI) than males (26.1 vs 25.0 kg/m^2^), while the reverse was true for blood creatinine and tHcy levels. The percentages of subject with diabetic fasting plasma glucose level (FPG ≥7.0 mmol/L) were 11.7% and 13.3%, respectively, in males and females. The percentages of subjects with CKD (eGFR < 60 ml/min/1.73 m^2^) were 3.1% and 3.4%, respectively in males and females. The risk-bearing *T* allele is more common than *C* allele, with an allele frequency of 0.51 and a *TT* genotype frequency of 0.26. The distribution was in Hardy-Weinberg equilibrium (p > 0.05).

**Table 1:** Phenotypic characteristics of study participants

**Variables**	**Male (N = 6914)**	**Female (N = 11900)**
Age, yrs	60.2 ±7.7	59.2 ± 7.5
BMI, kg/m^2^	25.0 ± 3.3	26.1 ± 3.7
SBP, mmHg	166.4 ± 20.9	169.1 ± 20.8
DBP, mmHg	97.1 ± 12.3	94.0 ± 11.5
Homocysteine, μmol/L	11.2 (9.3-14.2)	9.4 (7.8-11.6)
FPG, mmol/L	5.4 (4.9-6.2)	5.5 (4.9-6.2)
TC, mmol/L	5.2 (4.5-5.9)	5.4 (4.7-6.2)
Creatinine, mmol/L	71.0 (61.7-81.7)	56.3 (48.6-66.0)
eGFR, ml/min/1.73 m^2^	96.4 (87.9-104.2)	97.2 (87.0-104.5)
Current smoker, %	55.3	4.8
*MTHFR C677T*, %		
*CC*	23.9	23.5
*CT*	49.3	50.2
*TT*	26.8	26.2

*BMI* body mass index, *SBP* systolic blood pressure, *DBP* diastolic blood pressure, *FPG* fasting plasma glucose, *TC* total cholesterol, *eGFR* estimated glomerular filtration rate, *MTHFR* methylenetetrahydrofolate reductase. Age, BMI, and blood pressures were given by mean ± sd, serum markers were given by median (Interquartile range, IQR).

**Figure 1:**
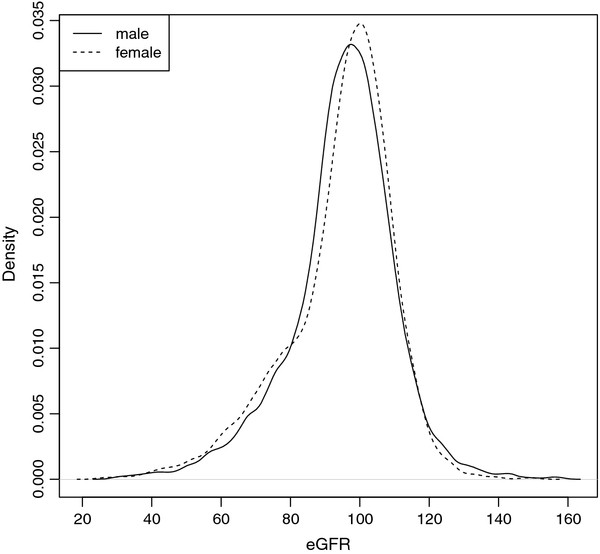
**The density curves of eGFR.** eGFR = estimated glomerular filtration rate, eGFR was in unit of ml/min/1.73 m^2^

The distributions of eGFR in both genders were quite similar, though it was slightly shifted to the right in female (Figure 1). Homocysteine and eGFR were inversely correlated, with spearman correlation coefficient rho = −0.25 (−0.22 in males and −0.28 in females).

Taking eGFR as a quantitative outcome in the regression analyses with adjustment for age, BMI, and systolic and diastolic blood pressure, a significant association of *MTHFR C677T* genotype and eGFR was observed in males but not in females (Table 2). In males, while there was little difference in eGFR among subjects with *CC* and *CT* genotypes, *TT* homozygotes had 1.37 ml/min/1.73 m^2^ lower eGFR than *CC* homozygotes (p = 0.004). Similarly, when dividing subjects in eGFR quintiles and comparing genotype frequencies, higher *TT* genotype frequencies were observed in the low eGFR quintiles (Q1-3, Table 3) in males. In females, genotype frequencies varied little across eGFR quintiles. In males, *TT* genotype was associated with a higher risk for decreased kidney function after adjusting for age, BMI, and blood pressures, with OR = 1.32 (95% CI:1.08-1.62, p = 0.008) for the lowest quintile of eGFR, OR = 1.25 (95% CI:1.11-1.41, p = 0.0003) for the lower half of eGFR, and OR = 1.21 (95% CI: 0.89-1.63, p = 0.22) for CKD defined as eGFR < 60 ml/min/1.73 m^2^. No such association was observed in females.

**Table 2:** **Regression analysis of eGFR and *MTHFR C677T* genotype**

** *MTHFR C677T* genotype**			**Males**				**Females**	
	**Mean eGFR**	** *β* **	** *SE* **	** *P* **	**Mean eGFR**	** *β* **	** *SE* **	** *P* **
*CC*	95.5	-		-	94.7	-		-
*CT*	95.3	−0.17	0.42	0.68	94.3	−0.42	0.33	0.20
*TT*	94.1	−1.37	0.47	0.004	94.7	−0.19	0.37	0.61

*MTHFR* methylenetetrahydrofolate reductase, *eGFR* estimated glomerular filtration rate, *β* regression coefficient, *SE* standard error, eGFR was in unit of ml/min/1.73 m^2^. Regression was done with adjustment for age, BMI, systolic and diastolic blood pressures.

**Table 3:** ** *MTHFR C677T* genotype frequencies in eGFR quintiles**

**eGFR Quintiles**	**Male**					**Female**		
	**N**	** *CC* (%)**	** *CT* (%)**	** *TT* (%)**	**N**	** *CC* (%)**	** *CT* (%)**	** *TT* (%)**
Q1 (0-20%)	2371	22.6	48.5	28.9	1375	23.6	50.6	25.8
Q2 (20-40%)	2371	23.6	48.9	27.6	1375	22.9	50.4	26.7
Q3 (40-60%)	2371	23.0	48.7	28.4	1375	24.0	49.1	26.9
Q4 (60-80%)	2371	25.2	50.1	24.7	1375	21.9	51.3	26.7
Q5 (80-100%)	2372	25.0	50.5	24.7	1375	25.3	49.7	25.0

*MTHFR* methylenetetrahydrofolate reductase, *eGFR* estimated glomerular filtration rate.

Since C677T genotype, homocysteine, and eGFR are significantly correlated, it is difficult to segregate the individual effect of genotype and homocysteine on eGFR in a regression analysis. Nevertheless, regression analysis of eGFR with an interactive term between genotype and homocysteine (Table 4) revealed the similar sex-specific effect of C677T genotype, while plasma homocysteine levels were inversely associated with eGFR in both genders. A significant interaction between TT genotype and homocysteine on eGFR was observed only in males: the regression coefficient of homocysteine was attenuated in TT homozygotes compared with CC homozygotes (p = 0.002).

**Table 4:** **Regression analysis of eGFR with interaction between homocysteine and *MTHFR C677T* genotype**

	**Males**			**Females**		
	** *β* **	** *SE* **	** *P* **	** *β* **	** *SE* **	** *P* **
CT	−3.54	3.12	0.26	−0.83	2.30	0.72
TT	−9.39	3.16	0.003	−0.79	2.48	0.75
log(Hcy)	−6.73	1.15	5.7x10^-9^	−7.10	0.89	1.4x10^-15^
CT:log(Hcy)	1.52	1.32	0.25	0.42	1.04	0.69
TT:log(Hcy)	3.91	1.29	0.0024	1.04	1.08	0.34

Regression was done with adjustment for age, BMI, systolic and diastolic blood pressures (coefficients not shown). Log(Hcy) was the natural log of plasma homocysteine in μmol/L. *β* regression coefficient, *SE* standard error, eGFR was in unit of ml/min/1.73 m^2^.

## Discussion

In this report, we have demonstrated that *MTHFR C677T* genotype is associated with eGFR in Chinese males, but not in females. The association still holds after adjusting for age, BMI, and blood pressures.

The *Val* form of MTHFR encoded by the *677 T* allele is thermolabile and has reduced enzymatic activity [9]. The degree of enzyme thermolability (assessed as residual activity after heat inactivation) is much greater in 677TT individuals (18-22%) compared with *C677T* (56%) and *C677C* (66-67%). The allele frequency of *677 T* varies with populations, ranging from less than 10% in African to 50% in Chinese [12]. Consistent with previous reports, in our study samples *677 T* is the major allele with an allele frequency of 0.516 and *TT* homozygote frequency of 0.26.

Many cross-sectional studies showed that plasma tHcy and kidney function was negatively correlated. For example, in NHANES III study [1], the risk for CKD defined as eGFR < 60 ml/min/1.73 m^2^ in individuals with high tHcy level (>11umol/L) was 40 times higher than those with low tHcy level (<7umol/L). However, it’s still not clear if hyperhomocysteinemia is the true effector that leads to decreased kidney function or it is simply a marker for kidney function. A recent prospective study in the Framingham cohort demonstrated that baseline homocysteine is an independent risk predictor for CKD and urine microalbuminuria [8]. In animal studies, induced hyperhomocysteinemia could cause podocyte injury and glomerulosclerosis [5-7]. Feeding rats with methionine induced hyperhomocysteinemia and resulted in significant decrease of GFR.

In the current study, we explored association between *MTHFR C677T* and kidney function indexed by eGFR in a large Chinese sample, and found a significant association in males but not in females. In males, the association model was apparently recessive. While individuals with *CC* and *CT* had similar eGFRs, individuals with *TT* were associated with 1.37 ml/min/1.73 m^2^ lower value in eGFR, and an increased risk for decreased kidney function defined by bottom 20 or 50 percentiles. Of our study subjects, 3.3% had CKD with eGFR < 60 ml/min/1.73 m^2^, which is slightly higher than the 2.53% prevalence rate in China adults aged 35–74 years reported from the InterAsia [17], partly due to the hypertensive nature of our study sample. Our study also suggested TT genotype was associated with an increase risk of CKD (OR = 1.21, p = 0.22), though it didn’t reach statistical significance probably due to the low percentage of CKD in our sample.

We have also observed a significant interaction between C677T genotype and homocysteine on eGFR in males. In males, the apparent effect size of homocysteine on eGFR was significantly reduced in TT homozygotes compared with subjects with CC genotype. In other words, the TT effect size was bigger in subjects with lower homocysteine level. However, due to collinearity, it is difficult to segregate their effects under current study design. Another limitation of our study is the study population was hypertensive; the findings from this study may not be generalizable to general population. A confirmation in general population would be helpful.

## Conclusions

In conclusion, we have demonstrated *MTHFR 677 T* is a risk allele for decreased kidney function in hypertensive Chinese males, implicating this gene in the pathogenesis of CKD. Given the relationship among MTHFR, tHcy and eGFR, it would be temptational to speculate that tHcy mediate MTHFR’s effect on CKD. The ongoing CSPPT trial, which is essentially a homocysteine-lowering trial, should provide a more definitive answer when it completes.

## Abbreviations

tHcy, Homocysteine; MTHFR, Methylenetetrahydrofolate reductase; eGFR, Estimated glomerular filtration rate; CKD, Chronic kidney disease; BMI, Body mass index; FPG, Fasting plasma glucose level; CSPPT, China Stroke Primary Prevention Trial.

## Competing interests

The authors declare that they have no competing interests.

## Authors’ contributions

QD drafted the manuscript. YQC helped to draft the manuscript. XX performed data analysis. XX and FFF edited the manuscript. GFT, MLH, YFC, HXX, LMS, JPL and YZ assisted with data collection, coordination and oversaw sample collection. BYW, NLS, LSL, XPX, FFH, HBS and YH conceived of the study and participated in its design. All authors have read and approved the final manuscript.

## Pre-publication history

The pre-publication history for this paper can be accessed here:

http://www.biomedcentral.com/1471-2350/13/74/prepub
